# Homogeneously niobium-doped MoS_2_ for rapid and high-sensitive detection of typical chemical warfare agents

**DOI:** 10.3389/fchem.2022.1011471

**Published:** 2022-09-12

**Authors:** Huaning Jiang, Huaizhang Wang, Yanlei Shangguan, Jingfei Chen, Ting Liang

**Affiliations:** The Institute of NBC Defense, Beijing, China

**Keywords:** two-dimensional transition metal dichalcogenides, two-dimensional molybdenum disulfide, niobium doping, chemical warfare agents, sarin, sulfur mustard, gas sensor, room temperature

## Abstract

Rapid detection of Chemical Warfare Agents (CWAs) is of great significance in protecting civilians in public places and military personnel on the battlefield. Two-dimensional (2D) molybdenum disulfide (MoS_2_) nanosheets (NSs) can be integrated as a gas sensor at room temperature (25°C) due to their large specific surface area and excellent semiconductor properties. However, low sensitivity and long response-recovery time hinder the pure MoS_2_ application in CWAs gas sensors. In this work, we developed a CWAs sensor based on *in-situ* niobium-doped MoS_2_ NSs (Nb-MoS_2_ NSs) via direct chemical-vapor-deposition (CVD) growth. Characterization results show that the high content of Nb elements (7.8 at%) are homogeneously dispersed on the large-area 2D structure of MoS_2_. The Nb-MoS_2_ NSs-based CWAs sensor exhibits higher sensitivity (−2.09% and −3.95% to 0.05 mg/m^3^ sarin and sulfur mustard, respectively) and faster response speed (78 s and 30 s to 0.05 mg/m^3^ sarin and sulfur mustard, respectively) than MoS_2_ and other 2D materials at room temperature. And the sensor has certain specificity for sarin and sulfur mustard and is especially sensitive to sulfur mustard. This can be attributed to the improvement of adsorption properties via electronic regulation of Nb doping. This is the first report about CWAs detection based on two-dimensional (2D) transition metal dichalcogenides (TMDs) sensing materials, which demonstrates that the high sensitivity, rapid response, and low limit of detection of 2D TMDs-based CWAs sensor can meet the monitoring needs of many scenarios, thus showing a strong application potential.

## Introduction

Sarin and sulfur mustard, as typical Chemical Warfare Agents (CWAs), have the characteristics of easy preparation, easy storage, strong lethality, and a wide application range. Enemy easily uses them as weapons in wartime and peacetime. Injury incidents are not uncommon. Therefore, the rapid and accurate detection of CWAs is of great significance to maintain social stability and protect people’s lives and property. At present, we have made great progress in the research of CWAs sensors. The most widely used is the ChemPro100 sensor, which can classify and identify different CWAs based on open ion mobility spectrometry. The lower detection limit for Nerve Agents is 0.1 mg/m^3^, for Erosive Agents is 2 mg/m^3^, and for Systemic Agents is 50 mg/m^3^, and the alarm time generally does not exceed 30 s (Puton and Namieśnik, 2016). But there is still room for improvement in sensitivity and accuracy. It is crucial to develop a sensor with more excellent performance for CWAs.

As a new class of 2D TMDs, MoS_2_ has been widely studied and applied in the fields of lubrication, catalysis, optoelectronics, sensors due to its large specific surface area, high active site, suitable band gap, and unique semiconductor properties (Hwang et al., 2011; Liu et al., 2012; Butler Sheneve et al., 2013; Chhowalla et al., 2013; Huang et al., 2015a; Li et al., 2021; Zhang et al., 2021; Luo et al., 2022). Perkins et al. (2013) demonstrated that there has a certain gas-sensing performance for triethylamine (TEA), a decomposition product of Nerve Agents, of monolayer pure MoS_2_ NSs-based gas sensor, and it can detect to a minimum of 1 ppm TEA. Sarkar et al. (2015) proposed that doping metal can improve the gas-sensing performance of MoS_2_ NSs. They found that, by reducing the thickness of the MoS_2_ NSs and increasing the doping amount of metal atoms, the sensing performance of the MoS_2_ NSs-based gas sensor will gradually improve. We have prepared pure MoS_2_ NSs by CVD before and tested the gas-sensing performance of its based gas sensors for sarin and sulfur mustard (Wang Y. et al., 2020; Wang H. et al., 2020). The experimental results show that the pure MoS_2_ NSs-based gas sensors also have a certain gas-sensing performance for sarin and sulfur mustard at room temperature. But they are easily oxidized when exposed to air, and their carrier concentration and mobility are significantly reduced, leading to a sharp decrease in the gas-sensing performance (Barsan and Udo, 2001). Researchers found that by doping and compounding the pure MoS_2_ NSs, the original defects can be compensated and the proportion of pure MoS_2_ NSs bound to oxygen in the gas to be measured can be effectively reduced, which significantly improves the sensing performance of the material (Late Dattatray et al., 2013; Lee et al., 2013; Huang et al., 2015b; Liu et al., 2015; Yun et al., 2017; Qiao et al., 2018; Sharma et al., 2018; Zhou et al., 2018). Zhu et al. (2017) concluded that MoS_2_ NSs doped with Nb atoms are stable at room temperature. And they suggested that the doping of Nb atoms could significantly improve the adsorption properties, chemical activity, and sensitivity of the nanosheets to gas molecules due to the strong overlap between the 4d orbital of Nb and the outer electron cloud of gas molecules. Ivanovskaya Viktoria et al. (2008) systematically investigated the electronic properties of Nb atoms doped into MoS_2_ NSs by Density Functional Theory. They found that the doping of Nb into MoS_2_ NSs causes the nanosheets to generate electron holes and the MoS_2_ NSs will have the properties of metallic materials, such as enhanced material stability and faster electrical conductivity. Chen et al. (2017) found by calculation that S-Mo in MoS_2_ NSs is mainly ionic bonding, however, S-Nb is a mixture of ionic and covalent bonding together, and the delocalization is weaker than S-Mo bonding. Consequently, the stability of MoS_2_ NSs after doping with Nb is better than before. But it is very difficult to obtain MoS_2_ NSs homogeneously doped with metal. There are three main reasons, one is the doping of Nb atoms into MoS_2_ NSs, mainly by replacing the Mo atoms in the nanosheets with Nb atoms, the replacement work is more difficult, and uniform replacement cannot be guaranteed because of the chemical bond between S-Mo itself. Secondly, pure MoS_2_ NSs have defects, and it is easier to dope into Nb atoms at the defective positions. Thirdly, the nanosheets still have a certain thickness, and the Nb doping in different layers will be uneven by traditional methods such as electrostatic doping or dopant adsorption (Ivanovskaya Viktoria et al., 2008; Dolui et al., 2013). According to the characteristics of the CVD method, nanosheets can be deposited and grown layer by layer. In this work, homogeneously Nb-doped MoS_2_ NSs were controllably fabricated by controlling the reaction conditions and deposition time. Then, we also tested the effect of different molar ratios of Nb doping amounts on the gas-sensing performance of Nb-MoS_2_ NSs. Based on previous research experience, few-layer Nb-MoS_2_ NSs (3-5 layers) with 7.8% Nb doping content were selected for research and evaluated the gas-sensing performance of the sensor based on Nb-MoS_2_ NSs to detect sarin and sulfur mustard. The response of two typical CWAs by the Nb-MoS_2_ NSs-based gas sensor was preliminarily explored, and the sensing mechanism was revealed.

## Materials and methods

### Gas sensor fabrication

**FIGURE 1:**
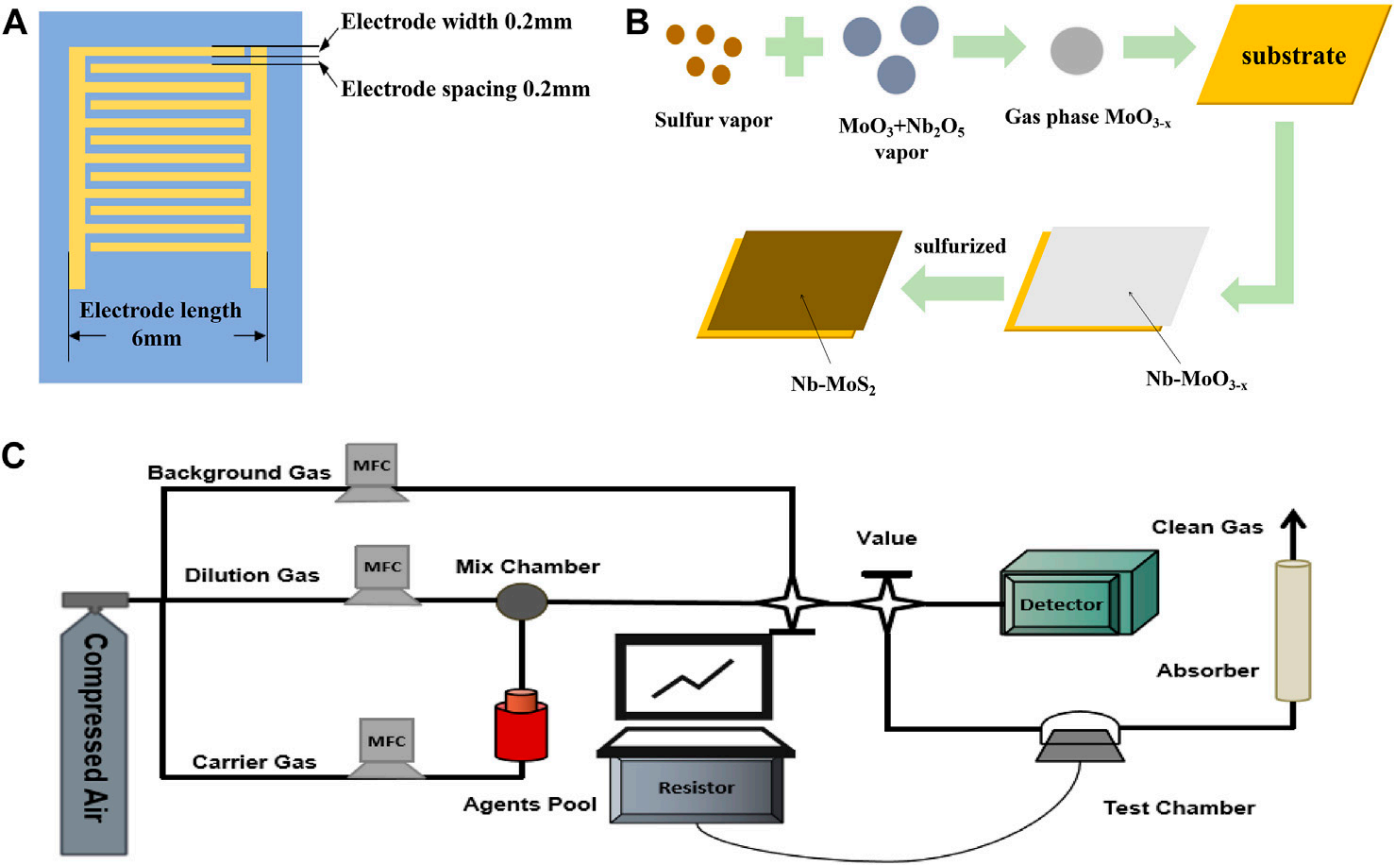
**(A)**The geometry of the electrodes of the sensor; **(B)** The process of fabricating electrodes and gas sensors, **(C)** A homemade dynamic gas detection system equipped.

The geometry of the electrodes of the gas sensor is shown schematically in Figure 1A. Twelve pairs of gold forked finger electrodes are processed on the Si/SiO_2_ substrate by the sputter coating method. The substrate area is 1 cm × 1 cm. The dimensions of the inserted finger electrodes are 0.6 cm × 0.6 cm × 0.01 cm (length × width × height), the width of each forked finger is 0.02 cm, and the spacing is 0.02 cm (Wang et al., 2013).

The process of fabricating electrodes and gas sensors is exhibited in Figure 1B. Firstly, argon was introduced into the tube furnace to achieve a vacuum atmosphere. Second, the temperature of the sulfur sublimation zone was controlled to be 180°C, the temperature of the deposition growth zone was 650°C, and the air pressure was 2000 Pa. 0.01 g Nb_2_O_5_, 0.03g MoO_3_ and 0.1g KCl were placed in a mortar and mixed by thorough grinding, and they were used as the Nb-doped Mo source. Finally, the solid sulfur powder was gasified at a high temperature as the sulfur source, and Nb-MoS_2_ NSs were deposited on the substrate.

The transfer was assisted using Polymethyl Methacrylate (PMMA). Firstly, a PMMA film was spin-coated on the as-grown Nb-MoS_2_ NSs. Secondly, the sapphire was etched with an appropriate amount of KOH solution to obtain Nb-MoS_2_ NSs (PMMA/Nb-MoS_2_) coated on the PMMA film. Then, the PMMA/Nb-MoS_2_ was transferred onto the prepared Si/SiO_2_ substrate engraved with 12 pairs of gold finger electrodes, between the substrate and the nanosheets. Finally, the PMMA was etched away with an appropriate amount of acetone solution, and the Nb-MoS_2_ NSs-based sensor was obtained.

### Experiment preparation

A homemade dynamic gas detection system equipped with gas distribution equipment and a data acquisition system was used to evaluate the gas-sensing performance, as illustrated in Figure 1C. The gas distribution lines were composed of the background gas, carrier gas, and dilution gas. Toxic gases are produced by purging the diffused sarin and sulfur mustard vapors from the agent pool with a carrier gas. The concentration of sarin or sulfur mustard passed into the test chamber is monitored in real time by a professional laboratory detector.

### Characterization

We characterize the morphology and structure of the nanosheets using scanning electron microscopy (SEM, ZEISS Gemini 300, Germany), transmission electron microscopy (TEM, FEI Strata 400S, Japan), and atomic force microscopy (AFM, Vecco Dimension 3100, Germany). Chemical compositions and valence states were analyzed using an energy dispersive X-ray spectrometer (EDS, 7426) and an X-ray photoelectron spectrometer (XPS, ESCALAB 250XI, United Kingdom). Raman spectroscopic analysis of the nanosheets was performed using a Raman microscope (Raman, Senterra & VeateX70, United Kingdom).

### Gas sensing measurements

All the related gas-sensing performances were tested at room temperature. Firstly, the sensor was placed in the test chamber which is equipped with an inlet and outlet pipe. Secondly, the background gas was purged into the test chamber to allow the sensing devices to reach a stable baseline (R_0_). Thirdly, the toxic gas concentration in the mixing chamber is controlled by adjusting the flow rate of carrier gas into the agent pool. Fourthly, the toxic gas concentration is adjusted by introducing dilution gas into the mixing chamber. Finally, the mixed gas is introduced into the test chamber for the experiment. The real-time electrical resistance (R) of the sensor was tested and recorded with Keithley Semiconductor Characterization Analyzers (DAQ6510, US). The response value is calculated by the change of resistance with the following formula:

$$Response=\left(R-R_{0}\right)*100\%/R_{0}$$ (1)

## Results

### Structural and morphological characteristics

**FIGURE 2:**
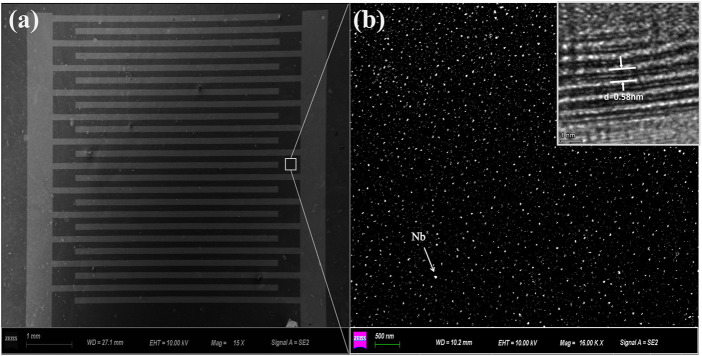
**(A)** SEM images of the Nb-MoS_2_ NSs (1 cm *1 cm) deposited on the surface of the substrate; **(B)** a highly uniform distribution of the agglomerated grains; the inset is a high-resolution TEM image of the lattice spacing.

The morphologies of Nb-MoS_2_ NSs were observed by SEM and TEM. The SEM image of the Nb-MoS_2_ NSs-based gas sensor is shown in Figure 2A. The distribution of the agglomerated grains is shown in Figure 2B. The Nb atoms show a highly uniform distribution, and the Nb-MoS_2_ NSs have a dense texture with no vacuum, cracks, or voids on the surface. As seen in the high-resolution TEM images in the inset, the Nb-MoS_2_ NSs are highly crystalline and possess an ordered lattice structure. The lattice spacing of the nanosheets is 0.58 nm (Late Dattatray et al., 2013), which is essentially the same as that of the pure MoS_2_ NSs. It indicates that the doping of Nb atoms does not change the lattice structure of MoS_2_ NSs, which is consistent with the existing reports in the literature that metal doping is atomic doping and does not change the crystal structure of 2D TMDs (Suh et al., 2014; Soo Yeon et al., 2015). This result is consistent with the finding of Dolui (Dolui et al., 2013).

**FIGURE 3:**
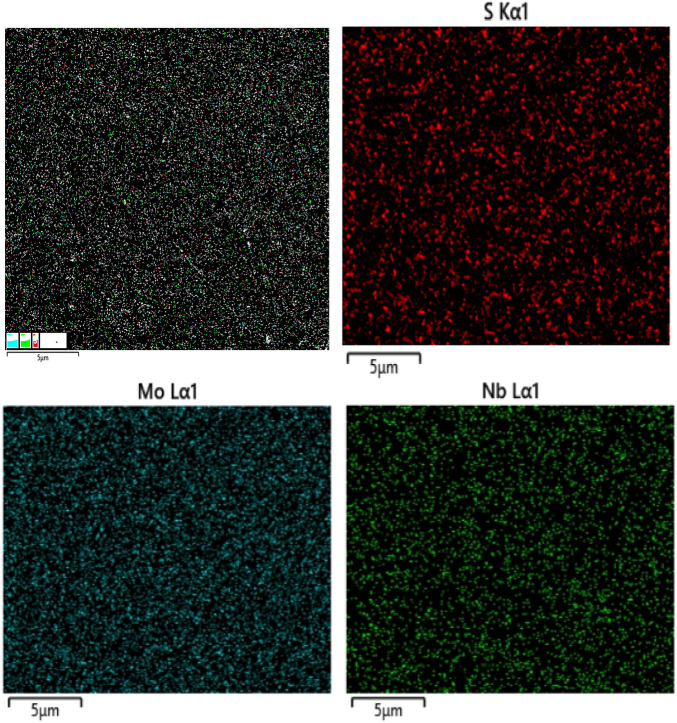
EDS elemental mapping.

Figure 3 exhibits EDS elemental mapping of S, Mo, and Nb acquired for the Nb-MoS_2_ NSs. The uniform doping of MoS_2_ by the Nb element in NSs was again verified. The measured atomic ratios are shown in Table 1. The doping ratio of Nb atoms is 7.80%.

**TABLE 1:** The atomic ratios of S, Mo and Nb for Nb-MoS_2_ NSs.

Element	Atomic, %
S	38.62
Nb	7.80
Mo	53.58
Totals	100.00

**FIGURE 4:**
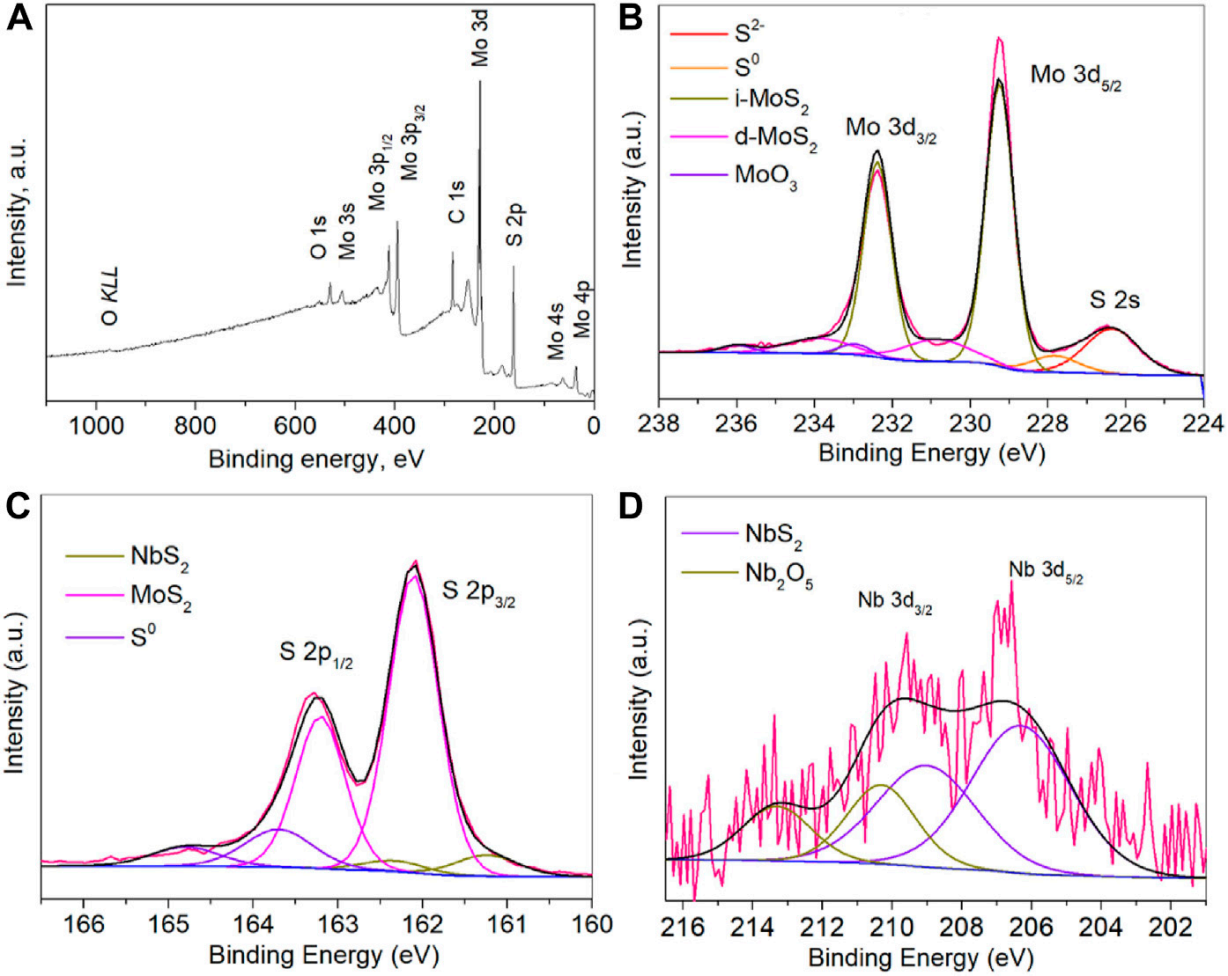
**(A)** XPS full spectrum of Nb-MoS_2_ NSs. A high-resolution scanning spectrum of the narrow region of **(B)** Mo 3d orbitals, **(C)** S 2p orbitals and **(D)** Nb 3d orbitals in Nb-MoS_2_ NSs.

Figure 4A shows the whole XPS survey spectrum of Nb-MoS_2_ NSs. The characteristic peaks of each major element in the nanosheets are readily visible in the figure and coincide with the XPS spectrum of MoS_2_. Figure 4B shows a high-resolution scanning spectrum of the narrow region of Mo 3d orbitals in Nb-MoS_2_ NSs. The native MoS_2_ (i-MoS_2_) peaks are located at 229.25 eV and 232.39 eV, while the surface-defective MoS_2_ (d-MoS_2_) of S cavities are located at 230.86 eV and 233.91 eV (Radisavljevic et al., 2011; Wang Y. et al., 2020). The double peaks of MoO_3_ are located at 232.96 eV and 236.01 eV, indicating the presence either of unreacted MoO_3_ or MoS_2_ which has been oxidized by air. There are also S 2s characteristic peaks present in the Mo 3d region. The one located at 226.35 eV is the characteristic peak of MoS_2_ and the one located at 227.84 eV is the characteristic peak of S monomers, considering that it may be due to the deposition of a small amount of unreacted S monomers. Figure 4C shows a high-resolution scanning spectrum of the narrow region of S 2p orbitals in Nb-MoS_2_ NSs. There are three sets of peaks in this part, those located at 161.24 eV and 162.34 eV are characteristic peaks of NbS_2_, and those located at 162.10 eV and 163.20 eV are characteristic peaks of MoS_2_, and those located at 163.68 eV and 164.78 eV are characteristic peaks of S monomers. Figure 4D shows the high-resolution scanning spectra of the narrow region of Nb 3d orbitals in Nb-MoS_2_ NSs. There are two sets of peaks in this part, those located at 206.31 eV and 209.01 eV are characteristic peaks of NbS_2_, and those located at 210.31 eV and 213.31 eV are characteristic peaks of Nb_2_O_5_, considering that it may be due to the deposition of a small amount of unreacted Nb_2_O_5_.

**FIGURE 5:**
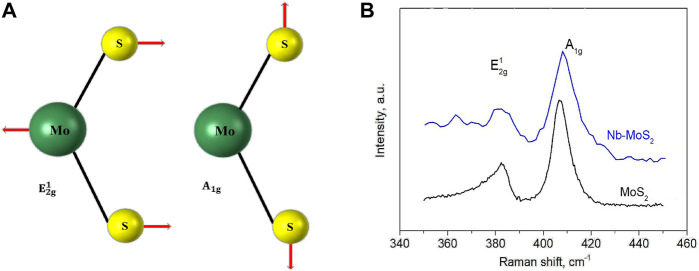
**(A)** Schematic diagram of the two vibrational modes $E_{2g}^{1}$ and $A_{1g}$ in the Raman spectrum of pure MoS_2._ **(B)** The Raman spectra of MoS_2_ NSs which were pure and Nb-doped.

As displayed in Figure 5A, two main vibrational modes the schematic diagram of the two vibrational modes ( $E_{2g}^{1}$ and $A_{1g}$ ) in the Raman spectrum of pure MoS_2_. The $E_{2g}^{1}$ mode originates from the vibration of two S atoms and Mo atoms in opposite directions along a two-dimensional plane, and the $A_{1g}$ mode originates from the vibration of S atoms along a direction perpendicular to the plane (Lee et al., 2012). Thus, Figure 5B shows two main characteristic peaks detected in the Raman spectrum of MoS_2_ NSs which were pure and Nb-doped. The Raman displacement difference between the pre-doping characteristic peak (383.8 cm^−1^ and 408.5 cm^−1^) is Δ = 24.7 cm^−1^, which is consistent with the characteristics of the few-layer MoS_2_ NSs. The Raman shift difference between the characteristic peaks (383.6 cm^−1^ and 409.2 cm^−1^) after doping is Δ = 25.6 cm^−1^, which is consistent with the characteristics of few-layer MoS_2_ NSs (Ganta et al., 2014). The two Raman characteristic peaks did not change much, and no characteristic peak of NbS_2_ appeared. It shows that the doping of Nb is atomic doping, and the introduction of Nb atom has little effect on the Raman shift of the in-plane vibrational mode and the interlayer vibrational mode of the S atom (Kim et al., 2014). It can be seen from the figure that the intensity ratio between the two Raman characteristic peaks weakened after the introduction of a small amount of Nb atoms. We can assume that the Nb doping weakens the S-atom intra-planar vibrational intensity and enhances the S-atom interlayer vibrational intensity (Ghatak et al., 2011).

### Gas-sensing performance

**FIGURE 6:**
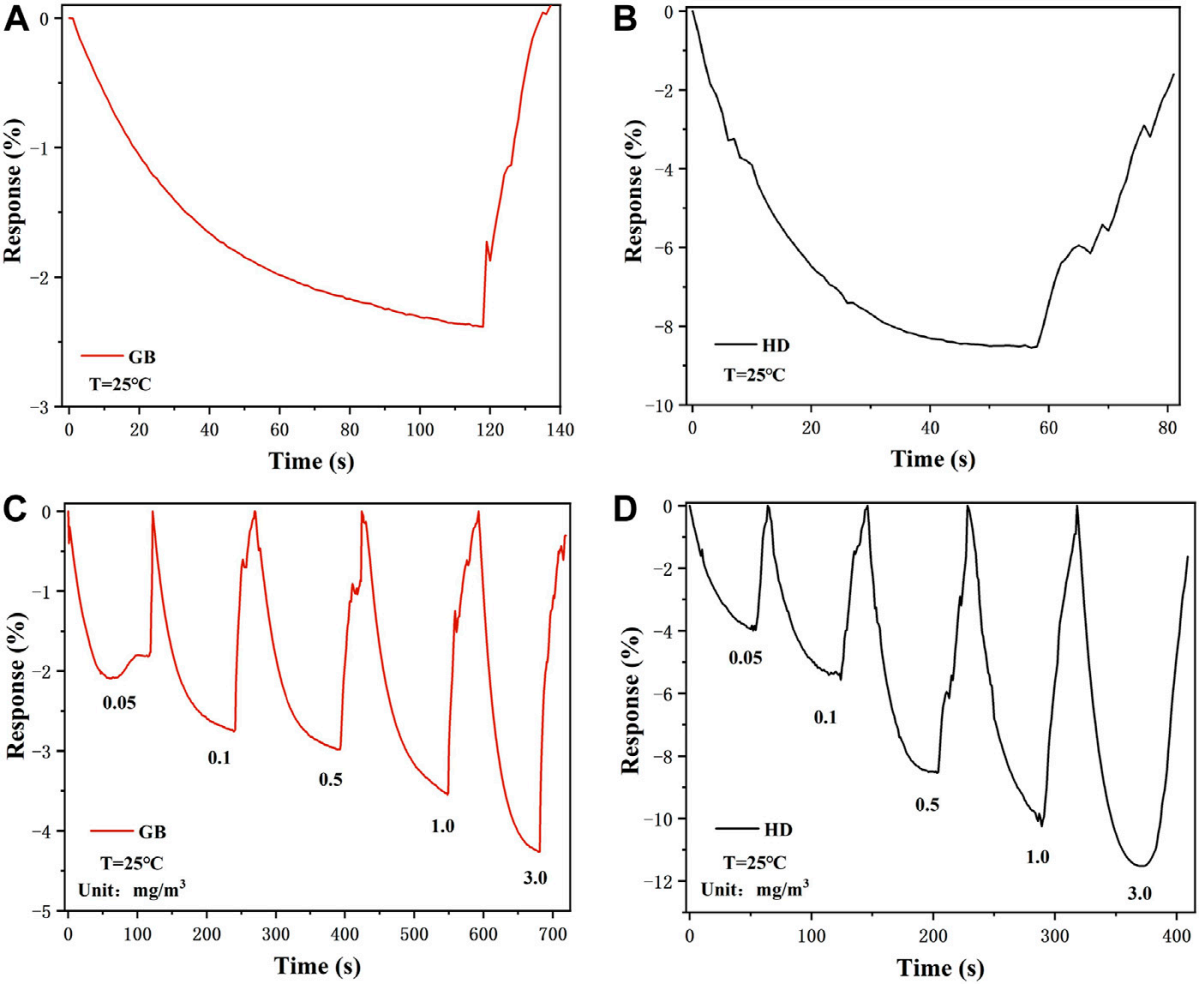
Response–recovery curve of the Nb-MoS_2_ NSs-based gas sensor to 0.5 mg/m^3^ **(A)** sarin and **(B)** sulfur mustard. Dynamic curves of exfoliated the Nb-MoS_2_ NSs-based gas sensor to different concentrations ranging from 0.05 to 3.0 mg/m^3^ **(C)** sarin and **(D)** sulfur mustard.

The long-term response curves of the Nb-MoS_2_ NSs-based gas sensor to 0.5 mg/m^3^ sarin and sulfur mustard are displayed in Figures 6A,B. When 0.5 mg/m^3^ of sarin was introduced into the test chamber, the response values of the Nb-MoS_2_ NSs-based gas sensors continued to increase until they reached a plateau at 120s. And the response time (T_90_, the time to reach 90% of the maximum response) is 78s. In the case of the same concentration of sulfur mustard being introduced, the sensor reached a plateau in the 60s, and the T_90_ is the 30s. This response time is much faster compared to other 2D TMDs-based gas sensors testing the same type of gas time (the other 2D TMDs-based gas sensors’ gas-sensing is shown in Table 2 shows). For the recovery process, the response value returns to its initial state within 30 s, which demonstrates the complete desorption of sarin and sulfur mustard from the Nb-MoS_2_ NSs-based gas sensor.

**TABLE 2:** Comparison of the CWAs sensing performance of Nb-MoS_2_ NSs-based senser with various types of 2D TMDs-based gas sensors reported in the literature.

Material	Gas	Response time (s)	Concentration (mg/m^3^)	Operating temperature (°C)	Response (%)	References
MoS_2_	TEA	10	0.06	25	−2.20	Butler Sheneve et al. (2013)
MoS_2_	Sarin	250	1.0	25	−2.01	Wang Y. et al. (2020)
MoS_2_	Sulfur mustard	316	1.0	25	−3.41	Wang H. et al. (2020)
V-MoS_2_	Sarin	500	0.02	25	−0.73	Wang Y. et al. (2020)
V-MoS_2_	Sulfur mustard	500	0.02	25	−2.13	Wang H. et al. (2020)
WSe_2_	DMMP	100	50.64	25	8.91	Li et al. (2020)
SnO_2_	DMMP	600	2.53	350	70	Soo et al. (2008)
rGO	DMMP	1080	101.29	25	8	Hu et al. (2012)
V-MoS_2_	Sarin	78	0.05	25	−2.09	This work
V-MoS_2_	Sulfur mustard	30	0.05	25	−3.95	This work

Furthermore, the dynamic response process of the Nb-MoS_2_ NSs-based gas sensor was recorded by exposure to various concentrations of sarin and sulfur mustard vapor ranging from 0.05 to 3.0 mg/m^3^, and the corresponding resistance variation curve is recorded separately in Figures 6C,D. The resistance of the Nb-MoS_2_ NSs-based gas sensor decreased gradually with the increase of sarin and sulfur mustard concentration. As the concentration of sarin increased from 0.05 to 3.0 mg/m^3^, the response changed from −2.09% to −4.26%. As sulfur mustard concentration increased from 0.05 to 3.0 mg/m^3^, the responsiveness changed from −3.29% to −11.52%. In addition, from Table 2, we can see that the gas performance of MoS_2_ NSs was significantly improved after doping with Nb atoms. The response value of Nb-MoS_2_ NSs to 1.0 mg/m^3^ sarin can reach −3.54%, which is 1.17 times higher than pure MoS_2_. As the response value of Nb-MoS_2_ NSs to 1.0 mg/m^3^ sulfur mustard can reach −10.25%, which is 2.71 times higher than pure MoS_2_.

**FIGURE 7:**
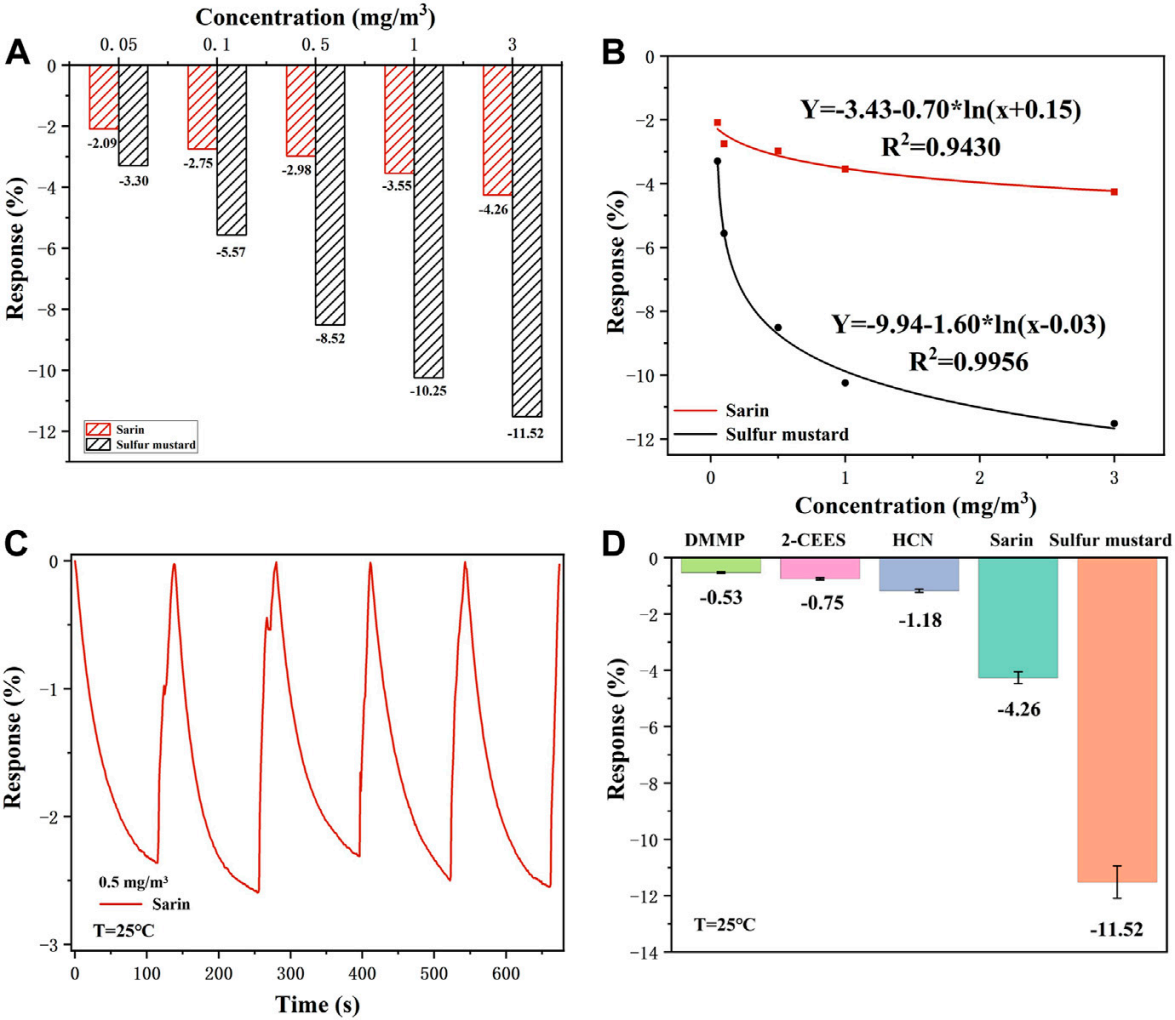
**(A)** The selectivity of the Nb-MoS_2_ NSs-based gas sensor for sarin and mustard in this concentration range. **(B)** A fitting equation between the response value and concentration of sarin and sulfur mustard. **(C)** Five successive sensing cycles of the Nb-MoS_2_ NSs-based gas sensor was continuously exposed to 0.5 mg/m^3^ sarin. **(D)** The selectivity of Nb-MoS_2_ NSs-based gas sensor to 3.0 mg/m^3^ and other analytes with 5 ppm concentrations.

As shown in Figure 7A, it is clear that the response value of the Nb-MoS_2_ NSs-based gas sensor to sulfur mustard is significantly higher than that of sarin, which indicates that the sensor is selective for sulfur mustard relative to sarin. It is reported that the difference in selectivity for organic vapors depends on the dipole movement (µ). The fitting equation of the response value and concentration of sarin and sulfur mustard, both of which follow the trend of logarithmic function distribution, is shown in Figure 7B. The charge transfer efficiency increases faster in the region of low sarin and sulfur mustard concentrations and increases with increasing gas concentration. However, when the concentration of both gases exceeded 0.5 mg/m^3^, the rate of resistance change started to decrease. Indicating that the active sites may eventually be occupied and the reaction reaches a state of relative saturation. In addition, repeatability and stability of a sensor are also a key performance indicator in practical sensing applications. Under the same conditions, the Nb-MoS_2_ NSs-based gas sensor was continuously exposed to 0.5 mg/m^3^ sarin, and response changes were recorded in Figure 7C (RSD = 5.07%). Similarly, Supplementary Figure S1 displays the long-term stability of the Nb-MoS_2_ NSs-based gas sensor that shows the response of the sensor remains nearly constant within five consecutive weeks, which confirms its excellent long-term stability.

To detect the selectivity of the Nb-MoS_2_ NSs-based gas sensor, the sensor is exposed to a series of potential interferents such as HCN, DMMP, and 2-CEES (all the potential interferent gas with a concentration of 5 ppm) under the same conditions. As shown in Figure 7D, when detecting CWAs and distractors, the sensor shows a big gap, and its responsiveness to CWAs is better, especially demonstrating optimum gas-sensing performance to sulfur mustard compared with other gases. Therefore, the Nb-MoS_2_ NSs-based gas sensor has the potential to serve as an outstanding candidate for sulfur mustard detection.

### Gas sensing mechanisms

Nb-MoS_2_ NSs are still n-type semiconductor materials. With the increase in Nb doping amount, the gas sensing performance of the Nb-MoS_2_ NSs-based gas sensor for both CWAs was improved. Because the pentavalent Nb^5+^ atom is mainly used as a donor impurity in MoS_2_ NSs, doped on the S-defect vacancy of MoS_2_ NSs. The surface of Nb-MoS_2_ NSs will form a donor defect-bound electron, which can transition to the conduction band of MoS_2_ NSs under excitation of lower energy, increasing the number of free electrons in the conduction band of MoS_2_ NSs. The electron density of Nb-MoS_2_ NSs surface is higher than that of pure MoS_2_ NSs. CWAs molecules are more likely to capture electrons on the surface of Nb-MoS_2_ NSs and form physical adsorption, thus causing resistance changes in Nb-MoS_2_ NSs (Nechita et al., 2011). During the response process, the donor impurity Nb indirectly reacts with the CWAs and makes its molecules adsorbed on the active spot on the surface of Nb-MoS_2_ NSs. It can be seen that Nb doping mainly improves the gas-sensing performance of NSs to CWAs molecules by increasing the active site of surface adsorption. In addition, Nb doping can inhibit the growth of grain size, decrease the grain size, increase the specific surface area and adsorption active site of nanosheets, and also improve the gas-sensing performance of nanosheets (Song et al., 2021).

**FIGURE 8:**
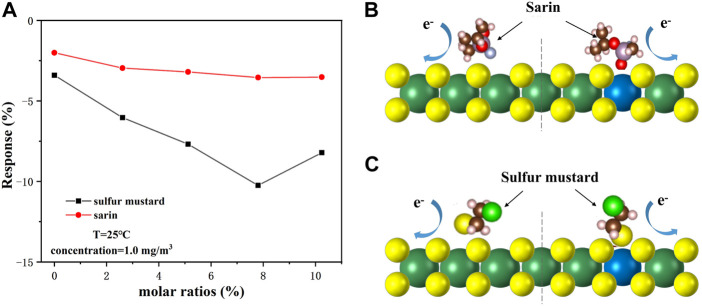
**(A)** Responses of different molar ratios of Nb-MoS_2_ NSs-based gas sensors to 1.0 mg/m^3^ sarin and sulfur mustard. Schematic diagram of the adsorption model of **(B)** sarin on MoS_2_ NSs before doping with Nb (left) and after (right), and **(C)** sulfur mustard on MoS_2_ NSs before doping with Nb (left) and after (right).

We also tested the effect of different molar ratios of Nb doping amounts on the gas-sensing performance of Nb-MoS_2_ NSs. Figure 8A shows that the gradual increase of Nb doping promotes the adsorption and dissociation reaction between the nanosheets surface and the CWAs molecules, thus improving the gas-sensing performance of the sensor. When the doping amount of Nb continues to increase to 10%, the Nb atom becomes an electron acceptor, which leads to the decrease of free electrons on the surface of nanosheets and the decrease in gas-sensing performance of nanosheets. However, the response of the Nb-MoS_2_ NSs-based gas sensor to sarin was less affected by the amount of Nb doping, while the response to sulfur mustard was more affected. We obtained the optimal sites and adsorption configurations of MoS_2_ NSs and Nb-MoS_2_ NSs for the adsorption of sarin and sulfur mustard by first-principles calculations, as shown in Figures 8B,C. As Nb doping changes the distribution of electrons on the surface of Nb-MoS_2_ NSs, the optimal adsorption sites and adsorption configurations also change. Because the sarin molecule is larger, and it is greatly affected by steric-hindrance effect when it adsorbs on the surface of Nb-MoS_2_ NSs, so the resistance of Nb-MoS_2_ NSs does not change significantly. However, the molecular volume of sulfur mustard is small, and it is mainly affected by the number of free electrons on the surface of Nb-MoS_2_ NSs during adsorption. Therefore, the resistance of Nb-MoS_2_ NSs changes significantly at this time. Based on the above analysis, we also consider that the Nb-MoS_2_ NSs-based gas sensor has better selectivity to sulfur mustard, which may be related to the fact that sulfur mustard molecules contain the same S atoms as those in nanosheets.

## Conclusion

In summary, we have reported a novel CWAs gas sensor based on 2D few-layer Nb-MoS_2_ NSs at room temperature. Homogeneously Nb-MoS_2_ NSs were controllably fabricated by CVD. Compared to pure MoS_2_ NSs, the CWAs-sensing performance of the Nb-MoS_2_ NSs-based gas sensor was significantly improved. The response value can reach −2.09% and −3.95% to 0.05 mg/m^3^ of sarin and sulfur mustard, respectively, with an exposure time of 78 s and 30 s. Moreover, the sensor displays reliable long-term stability and excellent selectivity. The improved gas-sensing performances are attributed to the increasing number of free electrons and the larger specific surface area, which provides more lone pair electrons and more active adsorption sites for CWAs molecules. Thus, the Nb-MoS_2_ NSs-based gas sensor with excellent performance is expected to become a novel device for room-temperature CWAs sensing applications. This is the first report about CWAs detection based on 2D TMDs sensing materials, which demonstrates that the high sensitivity, rapid response, and low limit of detection of 2D TMDs-based CWAs sensor can meet the monitoring needs of many scenarios, thus showing a strong application potential.

## Data Availability

The original contributions presented in the study are included in the article/Supplementary Material; further inquiries can be directed to the corresponding author.

## Supplementary material

Supplementary Figure S1 Long-term stability of the Nb-MoS_2_ NSs-based gas sensor to 0.5 mg/m^3^ sarin for 5 weeks.
